# Toxicity and Appeal of Flavoured E-Cigarettes and Flavour Ban Outcomes: A Narrative Review

**DOI:** 10.3390/ijerph23040416

**Published:** 2026-03-25

**Authors:** Stijn Everaert, Filip Lardon, Eric Deconinck, Sophia Barhdadi, Dirk Adang, Nicolas Van Larebeke, Greet Schoeters, Adrien Meunier, Veerle Maes, Suzanne Gabriels, Eline Remue, Katrien Eger, Pieter Goeminne, Frieda Matthys

**Affiliations:** 1 Chemical Environmental Factors Group, Superior Health Council, 1210 Brussels, Belgium; 2 Center for Oncological Research (CORE), University of Antwerp, 2610 Antwerp, Belgium; 3 Service of Medicines and Health Products, Sciensano, 1050 Brussels, Belgium; 4 Faculty of Medicine and Life Sciences, Hasselt University, 3590 Diepenbeek, Belgium; 5 Archaeology, Environmental Changes & Geochemistry, Vrije Universiteit Brussel, 1050 Brussels, Belgium; 6 Centre for Carcinogenesis and Primary Prevention of Cancer, Ghent University, 9000 Ghent, Belgium; 7 Department of Biomedical Sciences, University of Antwerp, 2610 Antwerp, Belgium; 8 Centre d’Aide aux Fumeur, Hôpital de la Citadelle, 4000 Liège, Belgium; 9 Kom op Tegen Kanker (Belgian Cancer Society), 1210 Brussels, Belgium; 10 Stichting Tegen Kanker (Belgian Foundation Against Cancer), 1030 Brussels, Belgium; 11 Directorate General Animals, Plants and Food, Federal Public Service Health, Food Chain Safety and Environment, 1210 Brussels, Belgium; 12 Department of Pneumology, Antwerp University Hospital, 2650 Edegem, Belgium; 13 Department of Respiratory Medicine, VITAZ Hospital, 9100 Sint-Niklaas, Belgium; 14 Department of Psychiatry, Vrije Universiteit Brussel (VUB), 1090 Brussels, Belgium

**Keywords:** e-cigarettes, nicotine, respiratory toxicity, flavourings, flavour ban, public health policy, precautionary principle

## Abstract

**Highlights:**

**Public health relevance—How does this work relate to a public health issue?**

- The rising prevalence of vaping among young never-smokers represents a serious public health concern.
- While flavours strongly increase the appeal of e-cigarettes to youths, flavouring substances contribute to the overall toxicity of vaping.

**Public health significance—Why is this work of significance to public health?**

- This work provides a highly multidisciplinary synthesis of the e-cigarette flavour issue, covering toxicity, attractiveness, and international regulatory responses.
- It reviews international flavour ban experiences to inform policymakers on best practices, expected outcomes, and potential pitfalls.

**Public health implications—What are the key implications or messages for practitioners, policy makers and/or researchers in public health?**

- Banning non-tobacco e-cigarette flavours is a key measure to reduce youth appeal, e-cigarette use and initiation.
- Post-ban, effective enforcement, sound legislation, and comprehensive tobacco control strategies are crucial to prevent illicit sales, industry circumvention, and substitution to smoking.

**Abstract:**

Background: E-cigarette use has risen sharply among young never-smokers, largely driven by the availability of several thousand appealing flavours. This narrative review synthesises evidence on the health effects of vaping, flavour toxicology and attractiveness, designs and outcomes of flavour bans, and complementary measures. Methods: Peer-reviewed publications and institutional reports (up to January 2026) were retrieved from PubMed, Web of Science, Google Scholar, and reference lists of included articles. Evidence from about 200 references was synthesised by a multidisciplinary working group. Results: Although flavouring substances are generally considered safe for ingestion, their inhalation toxicity remains uncertain. In vitro and in vivo studies have reported oxidative stress, inflammation, cytotoxicity, impaired ciliary function, transcriptomic changes, genotoxicity, and DNA damage. These findings—along with the strong youth appeal of fruit/sweet flavours, the inconclusive effects of flavours on smoking cessation, and persisting uncertainties—support banning non-tobacco e-cigarette flavours under the precautionary principle. Flavour bans can reduce e-cigarette use and initiation, especially among young adults, although partial substitution towards combustible cigarettes has been reported in some U.S. states. Policy success requires effective enforcement, prevention of industry circumvention, curbing cross-border sales, and closing regulatory loopholes—ideally at the international level (e.g., EU-wide). Conclusions: E-cigarette flavours may increase vaping toxicity and strongly appeal to youth, justifying flavour bans to prioritise youth protection. To maximise effectiveness, accompanying measures and sustained investment in tobacco prevention, youth education, and accessible evidence-based smoking cessation support are essential.

## 1. Introduction

Electronic cigarettes (e-cigarettes, vapes, or Electronic Nicotine Delivery Systems, ENDS) entered the global market around 2006 as a potentially less harmful alternative to tobacco smoking [1]. Their use, however, has grown rapidly among never-smokers, particularly young adults and adolescents [1,2,3,4,5,6,7]. In Belgium, daily use among 15- to 24-year-olds rose from 0.6% in 2018 to 6.3% in 2023–2024 [8]. In Flanders, 29% of 12- to 18-year-olds had tried vaping [9]. Only 53.9% of young e-cigarette users (15–24 years) reported a history of smoking, versus 88.5–95.7% of adults (25–64 years) [8]. Consequently, vaping has become a distinct practice among the youngest generations. The latter are particularly vulnerable and impressionable [10], as the prefrontal cortex—critical for impulse control and decision-making—continues to mature until age 25 [11]. Young vapers are three times more likely to start smoking (gateway effect) [12].

E-cigarette aerosols raise broader toxicological concerns beyond nicotine. They typically contain propylene glycol (PG), vegetable glycerin (VG), flavourings, thermal degradation and interaction products, and even metals released from the device [13,14,15,16,17,18]. While the total number has likely increased since then, more than 7700 unique e-liquid flavours were available online in 2014 [19]. In 2017, the Dutch market alone offered nearly 20,000 e-liquids with 250 unique flavour descriptions [20]. E-liquid flavours are made from complex mixtures of individual flavouring substances. While the latter are generally considered safe for oral consumption as food additives by the European Food Safety Authority (EFSA), their inhalation toxicity is poorly characterised [13,21,22]. However, studies show that several flavourings induce a multitude of toxic effects [1,6,7,15,21,22,23,24,25,26,27,28,29,30,31,32].

To reduce the appeal of e-cigarettes among nicotine-naïve youth, multiple jurisdictions (including EU countries, U.S. states, and China) have restricted or banned sales of flavoured e-cigarettes [33,34,35,36,37,38,39,40,41,42,43,44,45,46,47,48,49,50,51,52,53,54,55,56,57,58]. In the EU, the Tobacco Products Directive 2014/40/EU (TPD) allows member states to decide independently over e-cigarette flavour regulations. Following the advice of its Superior Health Council (SHC), Belgium’s Minister of Public Health announced in November 2025 his plan to adopt a similar national flavour ban [59,60].

This paper by members of the SHC reviews (1) the health effects of vaping and the toxicology of e-cigarette flavours; (2) the role of flavours in product attractiveness; (3) design and outcomes of existing flavour bans; and (4) complementary measures to maximise policy impact. It aims to stimulate evidence-based tobacco control policies, grounded in the precautionary principle and focused on youth protection.

## 2. Methodology: Working Group and Narrative Literature Review

### 2.1. Working Group Composition

A multidisciplinary working group was established, in accordance with Article 5.3 of the World Health Organization (WHO) Framework Convention on Tobacco Control (FCTC). The deontological committee of the SHC screened all members for potential conflicts of interest. Participants from national academia, institutes, hospitals, and cancer prevention NGOs contributed expertise in toxicology, pneumology, carcinogenesis, tobacco prevention/cessation, addiction psychology, and tobacco control policy.

### 2.2. Narrative Review

This study examines the public health impacts of flavoured e-cigarettes and international flavour bans. A narrative review approach was selected for its flexibility and suitability in synthesising broad, complex evidence and providing a detailed, nuanced description and interpretation beyond the scope of more narrow, tailored systematic reviews [61].

Evidence collection was carried out collaboratively by all co-authors, each contributing field-specific expertise. The collected evidence (about 200 references) was discussed and thematically integrated across multiple consultation rounds, achieving a critical, multidisciplinary assessment of recent literature with national expert consensus. This is the main strength of our approach. Research gaps and remaining uncertainties were identified while applying the precautionary principle in interpretation.

The variable methodology of narrative reviews is both their greatest advantage and limitation. While providing a comprehensive synthesis, their reproducibility is lower than in systematic reviews.

### 2.3. Literature Search, Selection, and Inclusion Criteria

Peer-reviewed publications and institutional reports published up to January 2026 were retrieved via PubMed, Web of Science, Google Scholar, and by screening reference lists of included articles. Due to the thematic diversity of this review, search terms strongly varied across subtopics. Common examples included *“e-cigarette”, “flavour”, “toxicity”, “health effects”, “respiratory effects”, “cardiovascular effects”, “carcinogenicity”, “genotoxicity”, “oxidative stress”, “inflammation”, “biomarkers”, “*in vitro*”, “*in vivo*”, “flavour appeal”, “flavour preferences”, “flavour attractiveness”, “smoking cessation”, “harm reduction”, “flavour ban”, “flavour restrictions”*. Search terms were combined using Boolean operators (AND/OR). Given the broad, multidisciplinary scope and collective data gathering by the working group members, the PRISMA protocol was not applied. Unlike systematic reviews—which typically extract and track exhaustive raw search results—our narrative approach did not track such lists, due to practical constraints from numerous subtopic-specific searches. This flexibility enabled working group members to directly identify and select relevant studies within their fields of expertise, then collaboratively combine and supplement them to address remaining data gaps or inconsistencies.

Inclusion criteria were tailored to subtopic importance and data availability. Meta-analyses and systematic reviews were prioritised, supplemented by experimental studies to illustrate more specific insights/statements. Studies from the past five years were selected preferentially, but not exclusively. Older studies were included if still highly relevant. Titles and abstracts were screened for relevance, followed by a full-text assessment of eligible articles. Findings were synthesised thematically, giving higher weight to studies with robust methodology and representative samples. Potential bias was reduced by including multiple independent or complementary sources wherever possible. All tobacco/vaping industry-funded studies were excluded to guarantee scientific independence.

Finally, 172 different peer-reviewed journal articles, 21 institutional reports, and 5 (congress) abstracts were included (more information in Supplementary Materials).

## 3. Results: Health Effects of Vaping and the Toxicology of E-Cigarette Flavours

### 3.1. General Health Impact of Vaping (Total Exposure)

**Figure 1:**
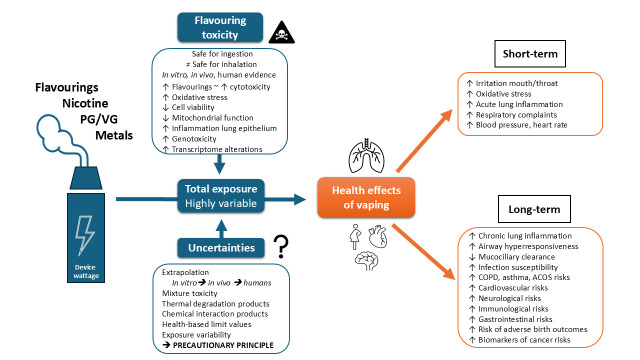
Summary of the health effects of vaping, flavourings, and existing uncertainties.

The health impact of vaping exposure has been treated in multiple reviews [1,5,6,7,62,63,64,65,66] and reports [4,14,60,67,68,69,70]. Although e-cigarette aerosols probably exhibit lower puff-for-puff toxicity than combusted tobacco smoke for cytotoxicity and lung inflammation [7], vaping is associated with respiratory and cardiovascular effects, alongside potential neurological, immunological, gastrointestinal, and perinatal effects [1,6,7,29,63,64,66,71,72,73,74,75,76,77,78,79,80,81] (Figure 1). Moreover, epigenetic changes via DNA methylation have been reported in multiple human tissues [78].

Short-term effects include acute mouth/throat irritation and acute respiratory complaints (including coughing, increased airflow resistance, asthma exacerbations) [5,7,13,66]. (Pro)inflammatory effects occur, including cytokine release by lung (epithelial) cells (in vitro/in vivo) [5,6,7,82] and by nasal epithelial cells (human data) [6,83]. Transcriptomic changes in small airway epithelium and alveolar macrophages have been documented in humans after acute exposure [84]. Similar to other nicotine products, blood pressure and heart rate increase shortly after use of nicotine-containing vapes [4,5,66], while vaping is also associated with an acute increase in arterial stiffness and endothelial dysfunction in humans [4,66,85,86,87]. Preclinical data suggest that acute exposure may accelerate the progression of certain cancers [1,88].

Long-term effects remain harder to study [6]. Chronic lung inflammation, reduced mucociliary clearance, disrupted airway epithelial barrier function, and impaired immune responses may exacerbate respiratory diseases, airway hyperresponsiveness, and increase susceptibility to respiratory infections [6,7,79]. Odds of self-reported asthma and asthma COPD overlap syndrome (ACOS) are elevated [81,89,90]. The same applies to COPD: recent meta-analyses suggest 1.46- to 1.50-fold higher COPD risks/odds among current e-cigarette users versus non-smokers (with varying adjustment for smoking history across studies; pRR 1.50, 95% CI 1.27–1.73; pOR 1.46, 95% CI 1.31–1.61; pOR 1.488, 95% CI 1.363–1.623) [81,91,92,93]. In comparison, COPD risk among current smokers remains 3.51 times that of non-smokers [94]. Moderate evidence exists for long-term cardiovascular risks [1,13,66], including myocardial infarction [95,96]. Pooled adjusted odds for developing cardiovascular disease were increased for e-cigarette users vs. non-users (pOR 1.24, 95% CI 1.05–1.46), and similar for e-cigarette vs. cigarette users (pOR 0.81, 95% CI 0.58–1.14) in a recently published meta-analysis [81]. In vitro findings suggest that chronic exposure may potentially increase bone resorption via reduced osteoblast viability [97]. Epidemiological associations with lung and oral cancer remain inconclusive, but vigilance is warranted because long latency periods may delay observable outcomes [7,98,99]. Although lower than in smokers, substantial evidence demonstrates that using e-cigarettes is associated with multiple cancer risk biomarkers [100,101,102]. These include markers identified in multiple study types:

- In vitro (oxidative stress, inflammation, cellular apoptosis, DNA damage and genotoxicity, epithelial-to-mesenchymal transition) [7,82,103,104,105,106];
- In vivo in rodents (oxidative stress, inflammation, DNA damage, and genotoxicity) [82,107,108,109,110,111];
- In humans (carcinogenic substances/metabolites in urine, oxidative stress, inflammation, DNA damage, and genotoxicity) [65,112,113,114,115,116,117].

### 3.2. Toxicity of Flavouring Chemicals and E-Liquid Flavours

Flavours arise from complex mixtures of flavouring substances; their number typically ranges between 1 and 50 per e-liquid bottle [28], with published means of 10 ± 15 [118] and 6 ± 4 flavourings per e-liquid [119]. Depending on their chemical composition, flavours may contribute to the toxicity of e-cigarettes [22,23,24,25] (Figure 1). Most evidence comes from in vitro exposures of human cells to individual flavourings or well-defined flavour mixtures [6,21,25]. While diluted liquids can be used with fixed concentrations, more realistic aerosol exposures are simulated with smoking/vaping machines and fixed puffing regimes [24,120]. With thousands of flavourings available, in silico predictive tools aid prioritisation for in vitro testing [15]. However, in vitro results cannot be directly extrapolated to living organisms. Simplified experimental setups may overestimate toxicity, for example, due to unrealistically high exposure without accounting for physiological barriers, catabolism, and aerosolisation losses of toxicants [121]. In vivo experiments with rodents approximate real-world exposure, including toxicokinetics [6,30,122], but introduce interspecies differences (different anatomy, physiology, and susceptibility) [22]. Studies in humans on the specific effects of flavour(ing)s are still largely lacking. Such studies exist on the effects of vaping in general, both observational [123] and mechanistic via molecular-epidemiological approaches like biomonitoring (biomarkers in urine, saliva, plasma) [65,112].

#### 3.2.1. Flavouring Substances (Individual Chemicals)

High-throughput screening (HTS) assays indicated that e-liquids containing more chemicals are likely to be more toxic in vitro [26]. In addition, e-cigarette refill liquids with higher flavouring concentrations were more cytotoxic in vitro [27,28]. Despite being approved as food additives, several flavourings are known to cause respiratory toxicity and inflammation. For example, diacetyl and acetylpropionyl, used in buttery flavours, can exert profound lung toxicity (*bronchiolitis obliterans* in exposed workers) and display in vitro neurotoxicity [22,27,98,124,125,126]. Cinnamaldehyde, key to cinnamon flavours, suppresses ciliary motility in bronchial epithelial cells through mitochondrial dysregulation, impairs respiratory innate immune cell function, exhibits cytotoxicity toward human embryonic stem cells, pulmonary fibroblasts, and monocytes, and induces DNA strand breaks in vitro [7,23,127,128,129]. Vanillin and acetylpropionyl showed in vitro cytotoxicity and dose-dependent IL-8 secretion in human monocytes [23]. Menthol provokes in vitro oxidative stress, proinflammatory signalling, decreases cilia beating frequency, decreases mitochondrial functioning, while facilitating inhalation through the activation of the TRPM-8 cold and menthol receptor [1,22,130,131,132]. Moreover, combinations of ethyl maltol, furaneol, maltol, ethyl vanillin, benzyl alcohol, and vanillin, often present at concentrations > 1 mg/mL, were correlated with higher cytotoxicity (in vitro MTT assay) in popular e-cigarette refill liquids [27]. A direct correlation was found between e-liquid cytotoxicity and ethyl maltol concentrations [27]. The latter is one of the most common flavourings in e-liquids, after vanillin [118]. Ethyl maltol was also found to be a promoter of free radical formation, similar to citrus–floral flavourings such as citral, dipentene, linalool, and piperonal [133]. In another study on refill fluids, Margin of Exposure (MoE) calculations indicated non-trivial cancer risks in some products for pulegone (minty flavouring) and estragole (anise-like flavouring) [28]. Similar findings were reported earlier for pulegone [134]. Despite prohibitions of substances with Carcinogenic, Mutagenic and Reprotoxic (CMR) properties, known genotoxicants like safrole, 2-furylmethyl ketone, and 2,5-dimethyl-4-hydroxy-3(2H)-furanone were recently still detected in e-liquids [15]. Other carcinogens, such as acrolein and formaldehyde, could be generated by thermal degradation and pyrolysis of flavourings, alongside thermal decomposition of propylene glycol and glycerol [13,16,98,135].

#### 3.2.2. E-Liquid Flavours (Mixtures of Chemicals in Commercial Products)

As flavouring substances can be combined in various ways, two brands of the same flavour can display highly different toxic responses [136]. Reactive Oxygen Species (ROS) generation in aerosols varies significantly among different flavours and identical flavours with varying nicotine levels [137]. Alterations of pro-inflammatory biomarkers, cytotoxicity, reduced cell viability, and metabolic activity are often reported (mainly in vitro) after exposure to flavours like cinnamon, menthol, strawberry, coffee, and tobacco [7,24,31]. DNA damage is reported from in vitro experiments with tobacco, apple, cherry, menthol/mint, and other sweet/fruity flavours [7,25]. In vitro cytotoxicity, inflammation (IL-8 release), senescence (elevated SA-β-gal activity), and dysregulated wound healing responses in pulmonary fibroblasts were reported from an e-liquid with coconut, vanilla, and cookie flavours [32]. Exposure of human aortic smooth muscle cells to cinnamon, menthol, and tobacco flavours induced cytotoxicity and inflammation, with cinnamon flavour and high power settings eliciting the strongest response [138]. In vivo nose-only exposure of mice to cherry- and tobacco-flavoured e-cigarettes induced lung inflammation in a sex-dependent manner [30]. Offspring can also be affected: prenatal exposure of mice to vanilla-flavoured e-cigarette aerosols resulted in sex-specific lung transcriptome alterations at birth, increasing asthma susceptibility [29].

### 3.3. Uncertainties Justify the Application of the Precautionary Principle

Device wattage, PG/VG ratio, and nicotine concentration strongly influence thermal degradation and chemical interactions, yet these parameters are often overlooked [16,17]. Coil temperatures are generally below 300 °C when saturated with e-liquid, but can reach temperatures above 1000 °C when dry [139]. Above electric power levels of 40 W, the generation of thermal decomposition products may increase exponentially [135]. In chemically unstable liquids, flavouring-PG adducts may form with unknown toxicological properties [140]. For example, vanillin PG acetals were more cytotoxic than vanillin at the highest concentration tested [141]. Flavouring substances, reaction and degradation products, and other e-liquid constituents may interact additively, synergistically, or antagonistically. Such interactions make cumulative risk assessment under real-life conditions particularly challenging due to the complexity of combined exposures [142,143,144,145,146,147].

Another uncertainty arises from the complex and often undisclosed composition of flavourings [19,20]. Under the Commission Implementing Decision (EU) 2015/2183, ingredients present at concentrations below 0.1% may remain confidential, while the composition of herbal extract flavourings can vary depending on the growing conditions of the source material [21]. A full chemical characterisation of these products requires GC–MS analysis of generated aerosols, often complemented with other analytical techniques, such as ICP-MS for elemental analysis [17].

Even after chemical identification, data on inhalation toxicology, toxicokinetics, and toxicodynamics remain scarce, and respiratory health-based guidance values are lacking for many flavourings. Furthermore, existing health-based (occupational) limits are difficult to apply to vaping exposure patterns, which involve intermittent high peaks followed by periods of zero exposure [13,148]. Exposure estimates vary considerably with individual puffing behaviours and device characteristics [13], necessitating the use of multiple, conservative exposure scenarios. Therefore, it is still complicated to provide comprehensive risk assessments. Different methods have been suggested, such as the Margin of Exposure (MoE) approach [13,28,33,134,149]. The MoE is calculated by dividing a reference point on an in vivo dose–response curve (e.g., NOAEL or BMDL from animal studies) by the estimated human exposure [150]. MoE ratios above a certain threshold are considered of low concern [13,150]. For substances with low human exposure and insufficient toxicological data, the threshold of toxicological concern (TTC) approach is used, which is based on the chemical structure of the molecule [33,151].

Considering these uncertainties, the specific vulnerability of youth, and the toxicological concerns outlined above, e-cigarette flavours are not compatible with the preventive principles of physical-chemical environmental hygiene [60,144,145]. Hence, the precautionary principle should unequivocally guide future flavour policies.

## 4. Results: Flavoured E-Cigarettes and Attractiveness

While Article 7 of the European TPD (2014/40/EU) prohibits “characterising flavours” in cigarettes and roll-your-own (RYO) tobacco, flavours remain a defining feature of e-cigarettes, with a wide range available on the market [20]. In the 2021 opinion of the Scientific Committee on Health, Environmental and Emerging Risks (SCHEER), requested by the European Commission, it was concluded that strong evidence exists that *“flavours have a relevant contribution for attractiveness of use of electronic cigarette and initiation”* [13]. These findings were also reported by the systematic review of Meernik et al. [152] and the overview of systematic reviews by Livingstone-Banks et al. [25]. The latter study, however, estimated the impact on vaping initiation to be inconclusive [25]. Overall, it is important to understand specific flavour preferences and appeal among both non-smoking youth and (current) smokers.

### 4.1. Attractiveness to Young People (Adolescents and Young Adults)

Young people are particularly susceptible to the appeal of e-cigarette flavours. Flavours increase product appeal, willingness to use e-cigarettes, susceptibility to initiate vaping (especially among adolescents), and decrease harm perceptions (especially sweet fruit and candy flavours) [1,13,25,152,153]. The tobacco and vaping industries have aggressively targeted young people by introducing flavours such as popcorn, candy, and bubblegum, evoking pleasurable associations with familiar, everyday products. During adolescence, this exposure coincides with heightened curiosity, a stronger propensity for risk-taking, and increased sensitivity to peer and sibling influences [1]. Vaping may act as a gateway to tobacco smoking in this group, but the influence of flavoured vs. non-flavoured e-cigarettes in this transition requires further study [12].

In Western countries, young people prefer fruity, non-tobacco flavours [152,153,154,155,156,157,158,159,160,161]. In 2023, European vapers (current and former) aged 15–24 used fruit (56% & 73%), candy (24% & 43%), menthol (39% & 36%), and tobacco (34% & 26%) flavours at least on a monthly basis [154]. The popularity of fruit flavours declines with increasing age, whereas the use of tobacco-flavoured e-liquids rises, reaching 46% among current users aged ≥55 years [154].

In Belgium, a 2023 *Stichting tegen Kanker* survey distinguished between 15- to 20-year-olds (n = 110) and >20-year-olds (n = 60). The youngest group aligns with Eurobarometer trends by showing elevated fruit (59% berries) and candy (16%) flavour use [157]. Unlike EU data, however, menthol use was much lower (7%) among the youngest vapers, while tobacco flavours only appeared among e-cigarette users > 20-years (27%) [157]. In 2023, similar low popularity of menthol (8.7%) and tobacco (0.8%) flavours was documented among U.S. 8th, 10th, and 12th graders (ca. 13–18 years) [161]. Despite the small sample size of the Belgian survey, the greater popularity of menthol and tobacco flavours among the >20-year-olds may suggest a possible association with their higher intention to vape for smoking cessation (88%, vs. 20% among 15-to-20-year-olds) [157]. However, this remains uncertain, as no statistical test was performed. Curiosity about the available flavours was mentioned more often by the youngest group as a reason to initiate vaping (38% vs. 20%), while a similar proportion of both age groups indicated that they might quit if their favourite flavour were no longer available (26% vs. 22%) [157]. Another Belgian survey, issued by *Kom op tegen Kanker* in 2024, questioned 12- to 26-year-old Flemish people about their reasons to initiate vaping (n = 1294) and tobacco smoking (n = 894). When asked about the moment of first e-cigarette use, dominant answers were curiosity (58%), peer-influence (39%), and specific curiosity about flavours (36%). “It seemed tasty” (35%) scored much higher as a reason to start vaping, compared to smoking (16%) [162].

Similar to the Belgian findings, younger age (15–24 years) was associated with a lower likelihood of using tobacco flavours in 2018 in a Finnish study (univariate model, tobacco flavour vs. other flavours: OR 0.20, 95% CI 0.06–0.69) [45]. Recently, a randomised crossover trial among 21-to-35-year old citizens of Miami (US) found that menthol vaping enhanced e-cigarette use experience compared with tobacco flavour (scores *p* < 0.05), posing higher risks to nicotine-naïve youth to initiate vaping [163]. The most popular flavour category, fruit, was associated with significantly higher self-reported e-liquid consumption among young people in Canada, England, New Zealand, and the USA (*p* = 0.001) [164]. Landry et al. concluded that fruit flavours are more likely to motivate young American adults (18–24 years) to initiate vaping compared to older adults; fruit flavours were more likely to be purchased by the youngest group (*p* < 0.001) [158]. However, a recent systematic review concluded that the current evidence on this topic remains limited and of only moderate quality, preventing firm conclusions [159].

### 4.2. Attractiveness for Smoking Cessation

E-cigarettes have been promoted as a safer alternative to combustible cigarettes within a “harm reduction” framework. Tobacco/vaping companies often employ this concept to encourage smokers to “make the switch” rather than achieve sustained nicotine cessation [165]. This seems to be confirmed in practice: a 2022 meta-analysis found that 70% (95% CI 53–82%) of successful quitters continued using e-cigarettes after 6 months or longer [166].

Nevertheless, vaping has become the most frequently used smoking cessation aid in many Western countries. In England (2023–2024), e-cigarettes supported 40.2% of quit attempts (vs. 17.3% over-the-counter Nicotine Replacement Therapy [NRT], 40.8% no aid) [167]; in Belgium (2023–2024), vapes were used in 23.7% of quit attempts by daily smokers (vs. 12.2% NRT, 58.5% no aid) [168]. In the English cross-sectional study, vapers had higher quit success (aOR 1.95, 95% CI 1.74–2.17) [167], consistent with randomised trials [169] and the November 2025 Cochrane review [170]. The latter estimated slightly more successful quit attempts with nicotine e-cigarettes (8–11 per 100) compared with NRT or non-nicotine e-cigarettes (both 6 per 100), and no support or behavioural support only (4 per 100) [170]. While the Cochrane review included many Randomised Controlled Trials (RCTs), two 2021 meta-analyses of real-world, observational studies showed no association between e-cigarette use and smoking cessation (pOR 0.97, 95% CI 0.67–1.40 and aOR 0.90, 95% CI 0.63–1.27; pOR 0.947, 95% CI 0.772–1.160) [171,172].

The potential role of e-cigarette flavours in smoking cessation outcomes remains unclear. According to four recent systematic reviews, findings are inconclusive due to a paucity of data, highly heterogeneous study definitions, and methodological limitations [25,173,174,175]. Some evidence indicates flavour switching during quit attempts and a context-dependent preference for sweet flavours [174]. Few independent studies link flavours to increased adult smoking cessation: Friedman et al. reported associations with vaping non-tobacco flavours (aOR 2.28, 95% CI 1.04–5.01) [176]. No causality was assumed, as the results may reflect pre-existing preferences among those trying to quit [176]. This possible explanation is consistent with a Dutch sensory study showing that sweet- and menthol-flavoured e-liquids are liked equally by young non-smokers and adult smokers, more than tobacco flavours [177]. A large British trial (n = 886), published after the aforementioned reviews, concluded that use of tobacco flavour is associated with a lower smoking cessation rate compared to other flavours (RR 0.56, 95% CI 0.35–0.89) [178].

## 5. Results: Objectives, Designs, and Outcomes of Flavour Bans

Several researchers advocate a ban on flavours due to the specific appeal of flavoured e-cigarettes to young never-smokers [33,60,152,153,158,160,177,179]. Countries worldwide have introduced such restrictions [33,34,35,36,37,38,39,40,41,42,43,44,45,46,47,48,49,50,51,52,53,54,55,56,57,58], providing insights to optimise future policy measures. In the EU, Finland was the first to implement a flavour ban in 2016, followed by others from 2020 onwards. To date, Finland, Lithuania, Hungary, the Netherlands, Slovenia, and Latvia have banned all e-cigarette flavours except tobacco, while Estonia and Denmark also permit menthol [33,34,43,44,45,46,54]. Since October 2022, China has banned all non-tobacco flavoured e-cigarettes [50,51], aligning with similar measures in Singapore, Thailand, and Hong Kong [51]. In the US, no federal ban exists, but the Food and Drug Administration (FDA) prioritised enforcement against all unauthorised flavours except tobacco and menthol in cartridge/pod-based e-cigarettes on 6 February 2020, while the sales of flavours in other devices (disposable and tank-based) continued [41,55]. Following the 2019 outbreak of EVALI (E-cigarette or Vaping product use–Associated Lung Injury), several states implemented temporary restrictions [41]. As of January 2026, seven states (Massachusetts, New Jersey, New York, Rhode Island, Utah, Maryland, California) and Washington DC have permanently banned non-tobacco flavours, with menthol exemptions in some (Utah and Maryland) [38,41].

### 5.1. Objectives and Side Effects of Flavour Bans

The principal objectives and potential undesired consequences of flavour bans have been summarised in Table 1. Restrictions must primarily envisage youth protection by decreasing the appeal for e-cigarette initiation [38,56]. Another target may be decreasing the appeal of long-term dual use. Compared to cigarette use, evidence shows that combining smoking and vaping increases the odds of several diseases (pORs: cardiovascular disease 1.23, 95% CI 0.99–1.54; stroke 1.26, 95% CI 1.06–1.50; metabolic dysfunction 1.22 95% CI 1.15–1.31; asthma 1.20, 95% CI 1.12–1.28; COPD 1.41, 95% CI 1.12–1.64; oral disease 1.27, 95% CI 1.15–1.39) [81]. In a recent case–control study on early-onset lung cancer in the US, the odds among dual users were 2.8 times higher than for smoking alone [180].

On the other hand, smoking cessation rates and substitution patterns must be monitored to detect potential undesired substitution towards combustible tobacco [35,36,38,40,41]. Another threat is industry circumvention by introducing new vape types [44], flavoured liquids without nicotine are being sold as foodstuffs [54], the introduction of flavoured accessories (including mouthpieces with flavour capsules, flavour beads) [181,182] or additives like the synthetic coolant WS-23 [183]. In addition, e-cigarette users may add potentially harmful aromas not intended for vaping or illegal flavourings to unflavoured e-liquids [33,184]. Illegal sales (online and offline) may surge [185,186], and consumers may purchase flavoured products via cross-border sales [34,41,45,51,55], requiring more effective enforcement [182].

### 5.2. Designs of Flavour Bans

Multiple EU countries use negative lists specifying additives (including some flavourings) that are not permitted in e-cigarettes and tobacco products, often representing further interpretations of Art. 7(6) of the TPD for tobacco products [46,187]. Such lists provide policymakers with a tool to respond to new toxicological evidence, but this process takes time because technical restrictions must be notified to the EU (TRIS notifications).

Flavour bans generally operate at the ‘flavour’ level, mainly relying on consumer and behaviour data rather than toxicology, as the chemical composition can vary substantially. Tobacco flavours are exempted in European, Chinese, and U.S. state bans, as tobacco flavours appeal more to adult smokers than to young never-smokers [45,177]. While some studies also propose menthol exemptions [152,158] (policy in Denmark, Estonia, Utah, Maryland), others explicitly recommend banning menthol to protect young people [153,161,163,177]. Based on cross-sectional surveys among U.S. pupils (2020–2023), Bae et al. [161] concluded that menthol-flavoured e-cigarettes may particularly attract young people vulnerable to frequent vaping and rural youth. WHO policy recommendations call for banning ingredients that facilitate inhalation in all nicotine products, consistent with Article 7(6d) of the EU TPD [132]. Belgian legislation prohibits all facilitators of inhalation in tobacco products and e-cigarettes (Article 4, §4, Royal Decree of 28 October 2016) [132].

Another approach is a positive, restrictive list specifying flavouring substances permitted in e-liquids, with all others prohibited. Since January 2024, only tobacco flavour is allowed in the Netherlands, based on 16 flavouring substances [33]. A systematic methodology was used to select these substances, using ingredient data extracted from the European Common Entry Gate system (EU-CEG) [33]. Selection criteria were applied covering prevalence of use in tobacco-flavoured e-liquids, chemical composition, flavour description, and potential health effects. After excluding CMR substances, MoEs were calculated for different exposure scenarios. A TTC approach was applied when a Point of Departure (PoD) could not be determined [33]. A similar restrictive list exists in China [33], while a proposal with 40 tobacco and 42 mint/menthol flavourings by Health Canada in 2021 is not yet implemented at the Federal level [188].

### 5.3. Outcomes of Existing Flavour Bans

#### 5.3.1. European Union

Despite the introduction of flavour bans in multiple member states, few results have been published. The Eurobarometer reports 43% support among EU citizens in 2023 for banning flavoured e-cigarettes (vs. 40% opposed, 17% do not know) [154]. Excluding “don’t know” responses, support peaks in Lithuania (79%), Ireland/Estonia/Netherlands (73%), and Finland (69%). Support is lowest in Italy (41%) and in the Czech Republic (40%) [154].

In Finland, current e-cigarette use (1–2%) ranks among the lowest in the EU [45,154]. In 2018, two years after the ban, past-year vapers used unflavoured (43%) or tobacco-flavoured (24%) e-cigarettes, while banned flavours (especially fruit) persisted (43%) [45]. Nicotine-free flavoured liquids, sold as foodstuffs, circumvented the ban via sales in e-cigarette shops and online sales [45,54]. The flavour ban did not prevent e-cigarette use for smoking cessation, while no findings were reported on undesired substitution to combustible tobacco [45]. Compared to never-smokers, daily smokers were much more likely to use tobacco e-cigarette flavours vs. other flavours (univariate model OR 12.23, 95% CI 1.47–101.55) [45]. Therefore, the Finnish researchers concluded that banning all non-tobacco flavours was justified to prevent vaping initiation among never-smokers, who typically dislike tobacco flavour [45]. Enforcement challenges persist due to the high number of products notified for market access, limited safety data, limited tobacco control resources, and the unwillingness of the industry to comply with stricter regulations, resulting in court cases [54]. While the e-cigarette regulations were associated with reduced vaping among youths, the use increased between 2021 and 2023 when new, mainly non-tobacco flavoured products targeting regulatory loopholes became available [44]. Compared to 2017, use of non-tobacco flavours was less likely in 2019 (OR 0.73, 95% CI 0.71–0.75), 2021 (OR 0.53, 95% CI 0.51–0.55), but more likely in 2023 (OR 2.26, 95% CI 2.19–2.32) [44].

In the Netherlands, nine months after the January 2024 flavour ban, a cross-sectional survey among 548 13- to 24-year-olds and 457 ≥25-year-olds (all pre-ban vapers) found reduced vaping in 39.5% and quitting in 22.4% of respondents, with no differences between both age groups (*p* = 0.48 & *p* = 0.06) [34]. Use of non-tobacco flavoured e-cigarettes decreased from 91.4% to 47.0% (*p* < 0.01), with these often being purchased abroad (35.6%). The use of unflavoured e-cigarettes slightly increased among youth (from 1.9% to 3.7%, *p* = 0.01), in contrast to tobacco flavours [34]. Only a minority (27%) of those who have quit vaping due to the flavour ban (22.4%) used a replacement [189]. Six percent of all survey participants (pre-ban vapers) initiated cigarette smoking due to the ban, but the overall cigarette use continued to decrease (−8 percentage points post-ban), and use of other nicotine products remained stable, suggesting no net harmful substitution [34,190]. More detailed insights will be published in the near future by the Dutch RIVM [190]. A side-effect of the Dutch ban is the increased illegal trade; in 2024, the Dutch Food and Consumer Product Safety Authority (NVWA) blocked 3.5 million flavoured products, and led to the recall of 800,000 more [182]. Violations were identified in 17% of inspections, and hundreds of online advertisements and posts were removed in cooperation with social media platforms [182].

#### 5.3.2. United States

Multiple U.S. behavioural surveillance studies show that flavour bans reduce e-cigarette use/initiation among young people, while substitution towards combustible cigarettes may reduce or offset public health gains.

A large cross-sectional survey among 376,963 individuals aged 18–29 years (2016–2023) identified a 3.6 percentage point (ppt) (95% CI −5.0 to −2.1 ppt) reduction in daily vaping and a 2.2 ppt (95% CI, 1.0 to 3.4 ppt) increase in daily smoking, associated with state restrictions [36]. Compared with the 2018 mean rates, this corresponded to an 80% reduction in daily vaping and a 22% increase in daily smoking [36].

Effects vary by age. A 2017–2023 cross-sectional study (n = 72,170) by Lin et al. [56] reported a 6.05 ppt (95% CI, −11.21 to −0.90 ppt) decrease in e-cigarette initiation among young adults (18–24 years) in states with flavour bans, a halving of the rate before the ban (10.9%, 95% CI, 8.3% to 14.1%) [56]. The effects were more pronounced among individuals with greater societal advantages (for example, young adults with annual household income ≥$50,000; −6.92 ppt, 95% CI −13.12 to −0.73 ppt) [56]. No significantly reduced initiation was observed among adults (≥25 years) and adolescents (12–17 years), probably due to illegal purchase by the latter, as sales are prohibited below the age of 21 [56]. Similarly, Saffer et al. [41] observed no effects among ≥25-year-olds, while trends among adolescents were inconclusive. Among young adults (18–24 years), 2–3 ppt vaping declines (*p* < 0.05) were possibly offset by a similar increase in smoking [41]. Using different statistical models and analyses, Cotti et al. [40] found robust evidence that flavour restrictions reduce short-run frequent and everyday e-cigarette use by 2–3 ppt among youths (<18 years). While this effect may weaken after two years, the opposite appeared among 18- to 30-year-olds [40]. Also these researchers noted undesired substitution [40]. Cheng et al. [38] found associations between flavour restrictions and reduced e-cigarette use: −6.7 ppt (95% CI −1.3 to −12.1 ppt) in young adults (2022) and −1.2 ppt (95% CI −2.0 to −0.4 ppt) among adults ≥ 25 years (2023); and increased cigarette use among youths (+1.8 ppt, 95% CI 0.7 to 2.9 ppt in 2021) and young adults (+3.7 ppt, 95% CI 2.2 to 5.2 ppt in 2021; +2.7 ppt, 95% CI 1.4 to 4.1 ppt in 2022; +3.2, 95% CI 0.9 to 5.5 ppt in 2023) [38]. In an online, national survey among 18- to 34-year-olds in 2021 (n = 1253), Tam et al. [35] found that the second most common response under a real-world local ban was switching to smoking, while it would be quitting all tobacco under a hypothetical federal ban.

Sales data (2014–2020) align with these trends. Statewide restrictions on non-tobacco- flavoured e-cigarette sales in New York, Rhode Island, and Washington were associated with 25.0–31.3% reductions in mean 4-week total e-cigarette unit sales, compared to states without restrictions [42]. In New York, sales data suggest that no undesirable turn to cigarettes took place after the implementation of restrictions on the retail sale of flavoured vaping products in 2020 [58]. Before the ban in New Jersey, sales of flavoured e-cigarettes declined, and the decline accelerated after the restrictions, then slowed by the second half of 2020, with a brief increase in cigarette and cigar sales immediately post-ban [53]. In December 2025, the CDC Foundation reported that states that only restrict flavoured e-cigarettes did not experience any long-term increases in cigarette sales (New York and Rhode Island, 2020–2025) [191].

Youth awareness remains low, limiting impact. In New York (2021–2022), only 0.9% understood the ban, while 64% of aware young vapers reported no behavioural change, underscoring the need for better education [49].

#### 5.3.3. People’s Republic of China

In China, a cross-sectional study found a 5.8% decline in young non-users’ intention to try e-cigarettes post-flavour ban, particularly among females and those exposed to advertising [57]. Despite the ban in 2022, flavoured e-cigarette use has persisted [50,51], highlighting the need for clearer communication on flavour restrictions and vaping hazards to enhance public support [51]. Similar to adaptations among young Americans [55], young Chinese vapers continued using flavours through illegal or alternative ways, used tobacco-flavoured e-cigarettes, quit vaping, or returned to combustible cigarettes [51]. Extending flavour restrictions to all tobacco products may prevent switching to other harmful products [51].

### 5.4. Future Flavour Ban in Belgium (European Union)

In Belgium, e-cigarette regulations are relatively strict (Royal Decree of 28 October 2016). Since July 2023, non-nicotine e-liquids have been subject to the same requirements as nicotine-containing e-liquids (modification by the RD of 7 November 2022). Also, e-cigarettes with attractive features (not useful for the functioning of the device) have been banned (modification by the RD of 7 November 2022). In January 2025, Belgium became the first EU country to ban disposable vapes (modification by the RD of 3 May 2024) [192]. A majority (60%) of Belgian citizens support a flavour ban for e-cigarettes, according to the 2023 Eurobarometer [154]. In 2022, the Belgian Superior Health Council (SHC) proposed a moderate approach to introduce a positive list of permitted flavourings [69]. In 2025, the Council revised its stance in light of rising use among young never-smokers, the appeal of non-tobacco flavours for this group, and growing toxicological concerns. It unanimously recommended an “*urgent and drastic reduction in the number of flavours available for e-cigarettes*”, but proposed two models: a Dutch-style ban or an extension with up to three additional flavours for smoking cessation (each with a positive list of permitted flavourings) [60]. In November 2025, the Minister of Public Health announced plans to implement a Dutch-style ban [59].

## 6. Discussion

E-cigarette flavours pose a complex dilemma for tobacco control policies. While they may appeal to adults attempting to quit smoking, they strongly attract nicotine-naïve youth, particularly through fruit and sweet flavours that are appreciated by both groups. This review underscores the complex balance between toxicological risks and uncertainties, youth appeal, uncertain effects on smoking cessation outcomes, and the design and effects of flavour bans.

Although e-cigarette aerosols probably show lower puff-for-puff toxicity than tobacco smoke [7], substantial evidence links vaping to harmful short- and long-term effects, including adverse respiratory, cardiovascular, immunological, neurological, gastrointestinal, and adverse birth outcomes [1,6,7,29,63,64,66,71,72,73,74,75,76,77,78,79,80,81] (Figure 1). Long-term use may increase the risk of disabling conditions like COPD [81,91,92,93], and potentially cancer [100]. Unknown chronic toxicity, dual-use hazards [81,180], and gateway and re-entry effects [12] may undermine the benefits of “harm reduction” [7]. Thus, vaping initiation among young never-smokers may substantially contribute to future healthcare burdens.

The ever-increasing number of e-cigarette flavours is highly concerning. Flavours consist of mixtures of flavouring substances, with a mean of 10 flavourings per e-liquid [118]. These flavourings—considered safe for ingestion but poorly characterised for respiratory exposure—add to the overall toxicity of e-cigarettes. In vitro and in vivo studies show that multiple flavourings and flavour mixtures can induce adverse effects such as oxidative stress, (pro)inflammation, cytotoxicity, suppressed ciliary motility, transcriptomic alterations, genotoxicity, and DNA damage [1,6,7,15,21,22,23,24,25,26,27,28,29,30,31,32]. Key uncertainties persist, including the toxicity of chemical interaction products (e.g., flavouring-PG adducts), thermal degradation (depending on device wattage, PG/VG ratio), cumulative mixture toxicity, and variable exposure patterns, warranting application of the precautionary principle for youth protection. The lack of transparency in ingredients and the remaining toxicological uncertainties contrast sharply with EU REACH’s stringent chemical safety requirements.

Flavours undeniably enhance the appeal of e-cigarettes, decrease harm perceptions, and stimulate vaping initiation and curiosity among young people, especially among adolescents [1,13,25,152,153]. Young age is associated with higher curiosity, risk-taking, and susceptibility to peer influence. Western adolescents and young adults are particularly attracted to fruit and sweet flavours, whereas preferences for menthol and especially tobacco increase with age and smoking cessation intention [152,153,154,155,156,157,158,159,160,161]. While vaping may serve as a gateway to smoking among youth, the role of flavoured vs. non-flavoured e-cigarettes in this transition remains uncertain [12]. In contrast, despite the popularity of e-cigarettes among smokers as a cessation aid [170], evidence on the impact of e-cigarette flavours on smoking cessation is inconclusive and heterogeneous [25,173,174,175]. While some recent studies suggest that non-tobacco flavours may aid quitting [176,178], these associations may reflect pre-existing sensory preferences.

Based on these findings, evidence supports banning non-tobacco e-cigarette flavours as a precautionary youth protection measure. Using a well-designed selection method based on prevalence, hazards, and risks, the Netherlands only permitted 16 flavourings to compose tobacco-flavoured e-liquids (January 2024) [33]. Nine months post-ban, vaping declined markedly (–39.5%), and many vapers have quit (22.4%), seemingly without net harmful substitution, as cigarette use continued to decrease [34,189,190]. Similar effects were reported from New York, but other U.S. states documented undesired substitution towards smoking, reducing or potentially offsetting public health gains [35,36,38,40,41]. Recent data, however, also showed decreasing cigarette sales in U.S. states with e-cigarette flavour bans [191]. Flavour bans significantly reduced use and e-cigarette initiation among young adults (−2 to −6.7 percentage points) [36,38,40,41,56], halving pre-ban initiation rates [56]. American adolescents were likely less affected by flavour bans, as legal purchasing in the U.S. is not permitted until age 21 [56]. However, Cotti et al. [40] did find 2–3 ppt reductions in short-run frequent and everyday e-cigarette use among youths (<18 years).

As Ollila [54] aptly noted, “*regulating flavours to protect youth is wise although not easy*”. Illicit sales surged post-flavour ban in every country studied, with many users still accessing prohibited flavours [34,35,50,51,54,182]. Additional risks include do-it-yourself mixing with potentially harmful substances [184] and illegal vapes, often marketed to youth via social media (SnapChat, TikTok, Instagram) [185,186]. Illegal vapes often contain prohibited flavours or even drugs like synthetic cannabinoids (“spice”), which are currently circulating in European schools [193,194,195]. Effective flavour bans require robust enforcement by border control and product safety authorities, including continuous monitoring and adequate penalties for non-compliant importers, retail outlets, online sales/advertising [182]. To close regulatory loopholes, counter industry circumvention, and curb cross-border sales, policies should be harmonised internationally as much as possible. This may involve a pre-approval process for vaping products at EU level [54] and an EU-wide ban on non-tobacco flavoured vaping products through revision of the TPD. Regulations should also cover flavoured accessories, ban non-flavoured additives that enhance inhalation (for example synthetic coolants) [132], and equate requirements for non-nicotine and nicotine e-liquids, as in Belgian legislation.

Broader measures beyond flavour restrictions remain essential, within a strategy of strong tobacco prevention, youth education, and accessible cessation support. Additional policy options include retail display bans, prohibiting disposable vapes, banning non-functional features that enhance appeal, standardised e-cigarette packaging with limited flavour descriptors [196], and package leaflets with pictorial warnings, similar to Canadian cigarette inserts [68,197,198,199]. Innovative approaches also merit consideration, such as a generational sales ban on all tobacco products [200]. The concept of “harm reduction” and the role of e-cigarettes in smoking cessation remain disputed among health authorities. Despite strong disapproval from the WHO [2], the European Respiratory Society [201], the European Society of Cardiology [202], and the International Pediatric Association [203], the British Royal College of Physicians [14] recommends promoting e-cigarettes as an effective smoking cessation tool. In Belgium, the SHC regards e-cigarettes only as a potential, temporary cessation tool, preferably under professional supervision [68]. A similar view is shared by ANSES in France [70]. Continued investment in youth prevention and wider access to other, evidence-based cessation aids such as NRT or varenicline are therefore crucial. Ultimately, the goal should be complete nicotine cessation rather than “switching” from one form of dependence to another, as promoted by the industry [165].

## 7. Conclusions

In recent years, e-cigarette use has sharply increased among nicotine-naïve, non-smoking youth, largely driven by the wide variety of appealing flavours. Increasing evidence links vaping to short- and long-term adverse health effects. While flavouring substances are generally considered safe for ingestion, their toxicity via inhalation remains uncertain. Multiple potential toxic effects have been reported from in vitro and in vivo experiments. Given these findings, remaining uncertainties, strong youth appeal, and inconclusive evidence on the impact of flavours on smoking cessation, banning non-tobacco e-cigarette flavours is substantiated under the precautionary principle. Population-level youth protection must take priority over individual preferences of adult smokers. Flavour bans can reduce e-cigarette use and initiation, but the sales of illegal vaping products and undesired substitution to combustible cigarettes must be prevented. Therefore, policy success depends on accompanying measures and sound legislation: effective enforcement, prevention of industry circumvention, curbing cross-border sales, and closure of regulatory loopholes—ideally at the international level (e.g., EU-wide via revision of the TPD). To maximise effectiveness, a broader strategy of sustained investment in tobacco prevention, youth education, and accessible evidence-based smoking cessation support is essential.

## Figures and Tables

**Table 1:** Conceptual overview of objectives and potential undesired effects of e-cigarette flavour bans.

	Objectives of Flavour Bans	Potential Undesired Effects of Flavour Bans
**Target**	↓ Appeal for young never-smokers (<25 years). ↓ Long-term dual use (smoking + vaping)	↓ Appeal as a smoking cessation aid for (adult) smokers.
**Behavioural impact**	↓ Experimentation and vaping initiation among young never-smokers. ↓ Long-term nicotine addiction. ↓ Gateway effect of vaping among youth.	↑ Continuation of smoking among potential switchers. ↑ Relapse/substitution to combustible cigarettes among vapers.
**Regulatory approach**	Clear legal framework on permitted flavours/flavourings. Alignment with the “characterising flavour” ban in other tobacco products (EU TPD).	Industry circumvention via novel additives (e.g., synthetic coolants) and flavoured accessories.
**Market impact**	↓ Flavoured e-cigarette sales. ↓ E-liquid heterogeneity.	↑ Sales of other tobacco products. ↑ Illicit market sales. ↑ Illegal internet sales (including social media). ↑ Cross-border sales. ↑ Consumption of illegal e-liquids. ↑ Do-It-Yourself e-liquid mixing with unsuitable aromas, food additives, and illegal flavourings.
**Health outcomes**	↓ Preventable nicotine addiction. ↓ Vaping-associated morbidity.	↓ Smoking cessation rates among adults.
**Monitoring**	Youth vaping prevalence/initiation. Dual-use trends.	Smoking cessation rates. Substitution patterns. Illicit market surveillance.

## Data Availability

No new data were created or analyzed in this study.

## Supplementary Materials

The following supporting information can be downloaded at: https://www.mdpi.com/article/10.3390/ijerph23040416/s1. Table S1: Information and classification of included references.

## Abbreviations

The following abbreviations are used in this manuscript:

ACOS	Asthma-COPD overlap syndrome
ANSES	French Agency for Food, Environmental and Occupational Health and Safety
aOR	Adjusted Odds Ratio
BMDL	Benchmark Dose Lower Confidence Limit
CI	Confidence Interval
CMR	Carcinogenic, Mutagenic and Reprotoxic
COPD	Chronic obstructive pulmonary disease
DNA	Deoxyribonucleic acid
EFSA	European Food Safety Authority
ENDS	Electronic Nicotine Delivery System
EU	European Union
EU-CEG	European Common Entry Gate system
EVALI	E-cigarette or Vaping product use–Associated Lung Injury
FCTC	Framework Convention on Tobacco Control
FDA	Food and Drug Administration
GC-MS	Gas Chromatography–Mass Spectrometry
HTS	High-throughput screening
ICP-MS	Inductively Coupled Plasma–Mass Spectrometry
MoE	Margin of Exposure
MTT	3-(4,5-dimethylthiazol-2-yl)-2,5-diphenyltetrazolium bromide
NGO	Non-governmental organisation
NRT	Nicotine Replacement Therapy
NVWA	Dutch Food and Consumer Product Safety Authority
OR	Odds Ratio
PG	Propylene Glycol
PG/VG	Propylene Glycol/Vegetable Glycerin ratio
PoD	Point of Departure
pOR	Pooled Odds Ratio
ppt	Percentage Point
pRR	Pooled Relative Risk/Risk Ratio
RCT	Randomised Controlled Trial
RD	Royal Decree
ROS	Reactive Oxygen Species
REACH	Registration, Evaluation, Authorisation, and Restriction of Chemicals
RIVM	Dutch National Institute for Public Health and the Environment
RR	Relative Risk/Risk Ratio
RYO	Roll-Your-Own
SCHEER	Scientific Committee on Health, Environmental and Emerging Risks
SHC	Superior Health Council of Belgium
TRIS	Technical Regulations Information System
TPD	Tobacco Products Directive 2014/40/EU
TTC	Threshold of Toxicological Concern
U.S.	United States of America
VG	Vegetable Glycerin
VOC	Volatile Organic Compounds
WHO	World Health Organization
